# Lipoprotein-Associated Phospholipase A_**2**_ Mass Level Is Increased in Elderly Subjects with Type 2 Diabetes Mellitus

**DOI:** 10.1155/2014/278063

**Published:** 2014-04-10

**Authors:** J. Fortunato, V. Bláha, J. Bis, J. Št'ásek, C. Andrýs, J. Vojáček, B. Jurašková, L. Sobotka, P. Polanský, M. Brtko

**Affiliations:** ^1^3rd Department of Internal Medicine, Metabolism and Gerontology, University Hospital Hradec Králové, Medical Faculty, Charles University, 50005 Hradec Králové, Czech Republic; ^2^1st Department of Internal Medicine, Cardioangiology, University Hospital Hradec Králové, Medical Faculty, Charles University, 50005 Hradec Králové, Czech Republic; ^3^Department of Immunology and Alergology, University Hospital Hradec Králové, Medical Faculty, Charles University, 50005 Hradec Králové, Czech Republic; ^4^Department of Cardiosurgery, University Hospital Hradec Králové, Medical Faculty, Charles University, 50005 Hradec Králové, Czech Republic

## Abstract

*Objective*. Lipoprotein-associated phospholipase A_2_ (Lp-PLA_2_) is extensively expressed by advanced atherosclerotic lesions and may play a role in plaque instability. We selected a group of elderly subjects that underwent transcatheter aortic valve implantation (TAVI) or balloon angioplasty (BA) and separated them into two groups, diabetic and nondiabetic, to compare the level of Lp-PLA_2_ mass between them. *Methods*. 44 patients aged 79.6 ± 5.6 years with symptomatic severe aortic valve stenosis underwent TAVI (*n* = 35) or BA (*n* = 9). 21 subjects had confirmed type 2 diabetes mellitus. Lp-PLA_2_ mass was measured using an enzyme-linked immunosorbent assay kit (USCN Life Science, China) before and 3 days after the procedure.* Results*. Lp-PLA_2_ mass was significantly elevated in this population (1296 ± 358 ng/mL before TAVI; 1413 ± 268 ng/mL before BA) and further increased after TAVI (1604 ± 437 ng/mL, *P* < 0.01) or BA (1808 ± 303 ng/mL, *P* < 0.01). Lp-PLA_2_ mass was significantly increased on the diabetic group before these interventions. *Conclusion.* Lp-PLA_2_ may be a novel biomarker for the presence of rupture-prone atherosclerotic lesions in elderly patients. Levels of Lp-PLA_2_ in diabetic patients may accompany the higher amount of small dense LDL particles seen in these subjects.

## 1. Introduction

Lipoprotein-associated phospholipase A_2_ (Lp-PLA_2_), also known as platelet-activating factor acetylhydrolase, is a Ca^2+^-independent, 45 kDa protein encoded on chromosome 6p21.2-12 [1] lipolytic enzyme produced by the monocyte-macrophage line cells and to a lesser extent by lymphocytes and mast cells. It catalyzes the hydrolysis of the ester bond at the* sn*-2 position of phospholipids to yield lysophosphatidylcholine and oxidized nonesterified fatty acids [2]. These molecules have atherogenic properties including endothelial activation, suppression of nitric oxide generation, and inhibition of cell migration and proliferation. Lp-PLA_2_ acts on oxidized phospholipids [3] and 70–80% circulates by being bound to low dense lipoprotein (LDL) (although only 0.1% of LDL particles contain Lp-PLA_2_) and 20–30% binds to high dense lipoprotein (HDL) [4]. Overexpression of the enzyme is seen at the necrotic core of atherosclerotic plaques prone to rupture, where there is induction of foam cell apoptosis by oxidized LDL [5].

Lp-PLA_2_ is hypothesized to contribute to the development of atherosclerosis through the propagation of inflammatory processes in the arterial intima [6]. Several characteristics of Lp-PLA_2_ suggest that this enzyme could be specifically relevant in people with diabetes. First, diabetic subjects have increased circulating levels of small dense LDL, potentially providing an efficient mode of transport of Lp-PLA_2_ to the intima, since Lp-PLA_2_ has apparent binding preference for small dense LDL [7]. Second, free radicals from oxidation of excess glucose may increase the oxidation of lipoproteins [8] and thereby increase levels of the substrate for Lp-PLA_2_. Third, diabetes is characterized by increased macrophage infiltration of the arterial wall. Lp-PLA_2_ is secreted by macrophages, although the actual level of Lp-PLA_2_ production within the artery wall is unclear [6]. Finally, in addition to diabetic and insulin-resistant conditions providing a milieu that would foster increased Lp-PLA_2_ concentration and activity, Lp-PLA_2_ could potentially perpetuate insulin resistence through the generation of free fatty acids and increased chemotaxis, both of which could lead to persistent chronic inflammation [9].

Transcatheter aortic valve implantation is an alternative procedure for aortic valve surgery which involves substantial risks for elderly diabetic patients with symptomatic aortic valve stenosis combined with severe comorbidities. To predict the surgery risk, there are several system scores, one of the most commonly used being the EuroSCORE system (reviewed in 2011), designed to predict 30-day mortality rates. Concerning prognostic biomarkers predicting one-year outcomes after TAVI or surgical aortic valve implantation, a recent study proposed malondialdehyde, a marker of oxidative stress as a promising tool. Elevated pre-operative levels were associated with the severity of the disease and with worse prognosis [10]. Currently, no data on the Lp-PLA_2_ involved in the management, prognosis, and risk assessment in this patient's group are available. This biomarker was not yet tested to our knowledge in other studies, taking into consideration aortic valve interventions in elderly diabetic patients.

Inhibition of Lp-PLA_2_ by darapladib has proven to diminish the size and probability of rupture of the atherosclerotic plaque [11].

## 2. Patients and Methods

In this study, a cross-sectional analysis on 44 geriatric patients aged 79.6 ± 5.6 years that had undergone TAVI or BA for the treatment of severe aortic stenosis was performed. The patients were admitted to the Cardioangiologic Clinic of the University Hospital of Hradec Králové, Czech Republic, between January 2009 and January 2011. The subject characteristics are described on Table 1. Two of these patients had had a BA before TAVI (the first patient 8 months before and the second patient 1 month before); one of them needed BA 3 months after TAVI due to a perivalvular leak. The sample was then further divided into diabetic and nondiabetic patients. The study was approved by the local ethical committee and all patients gave a written consent.

The levels of plasma Lp-PLA_2_ mass were obtained using an enzyme-linked immunosorbent assay (ELISA) kit (USCN Life Science, China) before and 3 days after the procedure. Intra-assay variations were <10%, and interassay variations were <12%. The detection range of the assay was 0.625–40 ng/mL. Fasting blood samples were obtained immediately before and then 3 days after the cardiac procedure from every patient. Blood was drawn and centrifuged immediately. The serum was then aliquoted and stored at −80°C. Total cholesterol, HDL, and triglycerides were measured by enzymatic methods; LDL was measured by routine methods. A *P* value less than 0.05 denoted the presence of a statistically significant difference. For statistical analysis the open source software PSPP (Free Software Foundation, Inc., USA) was used. For comparison of the Lp-PLA_2_ mass before and after the cardiac intervention, the paired *t*-test was used. The correlations between the levels of Lp-PLA_2_ mass and other variables were calculated using Pearson's correlation coefficient. To calculate the differences between 2 groups (diabetic and nondiabetic, TAVI and BA), independent (unpaired) *t*-tests were used.

We included a control group using 48 healthy subjects living at a nursing home, giving us a population with 82.9 ± 4.2 years of age, with a mean level of Lp-PLA_2_ mass of 788.23 ± 210.95 ng/mL. The age of the subjects within study groups did not differ significantly; moreover, we provided control values for age-matched healthy individuals, and thus normalization according to the age of the subjects was not included into the results.

Measurements are expressed as mean ± SD or as total number, with the exception of CRP (median [range]). BP is the blood pressure, TAVI is the transcatheter aortic valve implantation, DM is the diabetes mellitus, HDL-C is the high-density lipoprotein concentration, LDL-C is the low-density lipoprotein concentration, CRP is the C-reactive protein, Lp-PLA_2_ is the lipoprotein-associated phospholipase A_2_, and BA is the balloon angioplasty.

## 3. Results

With respect to in-hospital combined safety endpoints (i.e., all-cause mortality, major stroke, periprocedural myocardial infarction, life-threatening bleeding, major vascular complication, and acute kidney injury), 1 patient (3%) died due to a cerebrovascular event after TAVI. During the two-year follow-up, 5 patients (11%) deaths were noted (2 from stroke, 2 from infectious complications, and 1 in a car accident).

**Figure 1:**
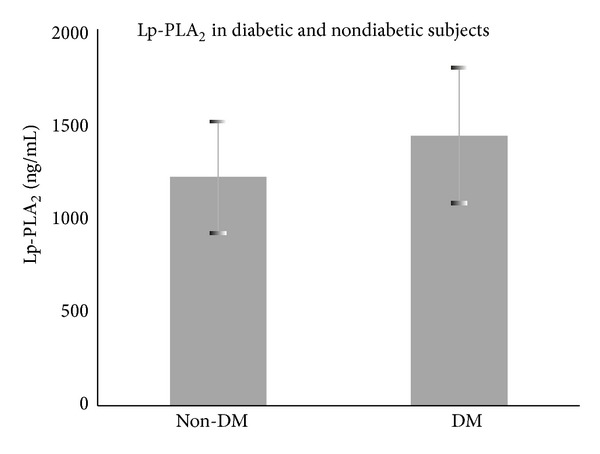
Lp-PLA_2_ mass was significantly increased on diabetic subjects.

The study population was then further divided into diabetic and nondiabetic patients to compare the levels of Lp-PLA_2_ mass between them. In the diabetic group, all patients were included without differentiation, if they were on diet, peroral antidiabetics, or insulin. Using the independent samples *t*-test, we concluded that the levels of Lp-PLA_2_ mass are statistically significantly increased (*P* = 0.03) in the diabetic group (1433.75 ± 353.58 ng/mL), when compared to the nondiabetic group (1215.7 ± 301.64 ng/mL) before any intervention was done (Figure 1). There were no statistical differences between the two groups concerning levels of total cholesterol, HDL, LDL, and triglycerides as well as BMI, and systolic and diastolic blood pressures. There were no statistical differences concerning the level of Lp-PLA_2_ mass after the cardiac procedure between the diabetic and nondiabetic groups.

Lp-PLA_2_ mass was significantly correlated with relevant cardiovascular risk factors (Table 2). As predicted, a strong correlation was found between Lp-PLA_2_ mass and LDL concentration (coefficient of 0.40, *P* = 0.01) as LDL is the main carrier for Lp-PLA_2_. The strongest obtained correlation was, however, found between Lp-PLA_2_ mass and triglycerides (coefficient of 0.43, *P* = 0.01). The enzyme mass was not correlated with body mass index (BMI) nor negatively correlated with HDL concentration.

**Figure 2:**
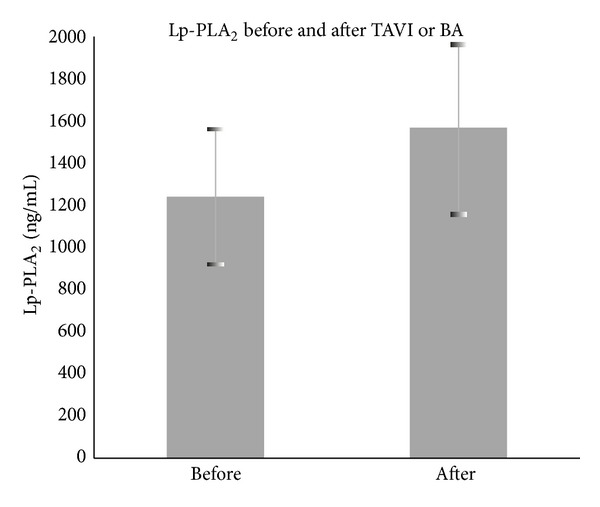
The levels of Lp-PLA_2_ significantly increased after manipulation with the aortic valve (both diabetic and nondiabetic groups are included for calculations).

It was then hypothesized that the levels of Lp-PLA_2_ mass rise after these cardiac procedures, TAVI (through either the apical or the transfemoral approach) and BA. Using paired *t*-tests we confirmed this increment, obtaining a *P* < 0.01 on both patient groups (1) for TAVI 1296 ± 358 ng/mL before the procedure and 1604 ± 437 ng/mL after it and (2) for BA 1413 ± 268 ng/mL before and 1808 ± 303 ng/mL after the procedure (Figure 2).

Furthermore, using the independent samples *t*-test we noticed that the levels of Lp-PLA_2_ mass before and after the procedure are not statistically significant when comparing it between the TAVI and BA groups (*P* = 0.36 and *P* = 0.15, resp.). This observation concluded that the enzyme mass significantly increased in the same proportion after manipulation with the aortic valve, regardless of the interventional method.

Finally, a control group of 48 healthy subjects was included to compare with the Lp-PLA_2_ mass before these cardiac interventions.

Using independent samples *t*-tests, we concluded that the mass of Lp-PLA_2_ is significantly increased in the study population (1319.77 ± 341.82) when compared to the control group (788.23 ± 210.95), *P* < 0.01.

## 4. Discussion

In the present study, we have shown that the levels of Lp-PLA_2_ mass, which are significantly increased in elderly patients when compared to normal population, further increase after TAVI or BA (treatment methods for severe aortic stenosis in high-risk patients). According to previous studies, the normal range for Lp-PLA_2_ mass concentration for men is 120–342 ng/mL (90th percentile) [12]. To date, many studies have been performed evaluating the role of Lp-PLA_2_ in atherosclerosis development. In fact, data from over than 50000 patients show an association between increased levels of activity, or mass of Lp-PLA_2,_ and an increased risk of cardiac death, myocardial infarction, acute coronary syndromes, and ischemic stroke [13]. The latter is of importance because LDL and other lipid factors have not been shown to be consistent predictors of stroke risk [14].

The levels of Lp-PLA_2_ mass increased after direct manipulation of stenotic aortic valves in elderly patients either through their replacement by TAVI or after providing BA. The most common cause of aortic stenosis is the age-related progressive calcification of a normal valve due to generalized atherosclerosis, with a mean age at presentation around 65–70 years. This biomarker was not yet tested to our knowledge in other studies taking into consideration aortic valve interventions. The impact of these results on the progression of atherosclerotic plaques and on the follow-up of patients with their valves operated on is still a topic for more discussion.

On the other hand, the association of Lp-PLA_2_ mass concentration with LDL, HDL, and total cholesterol has been established in several investigations [15–19]. Our results are in accordance with previous conclusions. As expected, Lp-PLA_2_ is positively correlated with LDL. Furthermore, it is correlated with total cholesterol and not with HDL.

Some controversy still exists whether Lp-PLA_2_ has pro- or antiatherogenic action. The later studies point towards a proatherogenic action in humans, once it is intrinsically related to LDL. However, in mice, it is mainly bound to HDL whereby it shows an antiatherogenic effect. For this reason, it can be assumed that the relative distribution of Lp-PLA_2_ may have an impact on its action, in other words, that Lp-PLA_2_ bound to LDL has a more proatherogenic action and Lp-PLA_2_ bound to HDL has a more antiatherogenic action.

Furthermore, we divided patients into diabetic and nondiabetic regarding the type of their antidiabetic therapy. 42% of diabetic patients were on diet, 14% on peroral antidiabetics, and 42% on therapy with insulin. From the diabetic patients, at the time of admission for the procedure on their aortic valve, 62% were being treated with a statin. Our study shows statistically significant increased levels of Lp-PLA_2_ mass in diabetic patients compared to nondiabetic (before any cardiac intervention). These observations were also shown in other studies [20, 21]. It is probable that significantly higher levels of total cholesterol, LDL, HDL, and triglycerides on diabetic subjects when compared to nondiabetic subjects were not found because many of the subjects (in both groups) were on statin therapy.

It is known that lysophosphatidylcholine in circulating LDL is increased in diabetic patients when compared with nondiabetic controls [22]. In a recent study, one more step was made regarding the role of lysophosphatidylcholine as an atherogenic molecule, when Iwase et al. [23] measured two major molecular species of this molecule and concluded that 1-palmitoyl lysophosphatidylcholine content in LDL correlated significantly with serum Lp-PLA_2_ levels in diabetic patients, but not 1-stearoyl lysophosphatidylcholine. In diabetic patients, there is a typical dyslipidemic triad of increased levels of LDL and triglycerides and decreased levels of HDL. LDL particles are smaller and denser, when compared to nondiabetics, and indexes of lipoperoxidation and negative electric charge of LDL are higher. Lp-PLA_2_ binds preferentially to small, dense, and electronegative LDL particles. One study evaluated the relative distribution of Lp-PLA_2_ in diabetic patients and concluded that, after improving LDL concentrations and glycemic control, the relative proportion of Lp-PLA_2_ bound to HDL (which had been suggested to be atheroprotective) increased [24].

In this study, there was no statistical difference concerning the triglyceride concentration between diabetic and nondiabetic patients despite a higher mean concentration on the diabetic group. However, in accordance with Noto et al. [20], there was a strong statistical correlation between Lp-PLA_2_ and triglycerides concentration. No other studies to our knowledge, apart from the referred above, have found this association. The occurrence of hypertriglyceridemia with low HDL cholesterol frequently seen in diabetic patients [25] promotes the formation of small dense LDL particles, prone to Lp-PLA_2_ binding. The underlying insulin resistance may impair the activity of lipoprotein lipase leading to decreased catabolism of triglyceride-rich Apo B lipoproteins [26].

Several limitations of our study should be taken into account. The first and most important drawback is the small number of subjects included in these investigations. The mean age of these subjects implies the coexistence of multiple comorbidities, some of which may influence the lipid metabolism. Moreover, accepted reference ranges for normal population are based on tests in adults. It is not known whether Lp-PLA_2_ mass increases with age independent of other factors. The current study suggests that the reference range for healthy elderly populations is much higher since the lowest mass level measured in the control group was 289.6 ng/mL. Despite the fact that all the laboratory analysis was performed under precise conditions, the Lp-PLA_2_ mass was approximately 5 times higher than the accepted normal range. The higher values observed in the present study may (a) reflect the widespread atherosclerosis levels seen in the elderly (supported by the mass levels found in the control group), (b) be a preanalytical effect of the storage of samples (despite all cautions and high standards of our laboratory), or (c) result from other, as of yet, undetermined factors.

## 5. Conclusion

Atherosclerosis is a complex chronic inflammatory condition. In the past years, a big effort has been made to prevent the associated cardiovascular events. Even with the use of statins, the levels of non-HDL cholesterol are not as desired. Lp-PLA_2_ is a promising marker to be included in the risk assessment for cardiovascular disease in the future. It can be considered a vascular-specific inflammatory marker, once its mass concentration or activity in the circulation only significantly increases after the enzyme is released from rupture-prone plaques. Other biomarkers such as high sensitivity CRP indicate a more nonspecific or generalized inflammation. Because diabetic patients are* per se* at increased risk to develop cardiovascular events, it would be also interesting to include Lp-PLA_2_ in the management, prognosis, and risk assessment in such patient's group. Direct inhibitors of Lp-PLA_2_ are currently undergoing clinical trials, and if they prove to reduce the necrotic core area of a rupture-prone plaque, they will solidify the importance of Lp-PLA_2_ as a therapeutic target for the management of atherosclerosis and prevention of myocardial infarction, stroke, and cardiovascular death.

## Figures and Tables

**Table 1:** Characteristics of subjects.

Total of subjects enrolled	44
Male	19 (43.2%)
Age (years)	79.6 ± 5.6
BMI (kg/m^2^)	27.6 ± 4.7
Systolic BP (mmHg)	133 ± 18
Diastolic BP (mmHg)	75 ± 10
Total patients after TAVI	35
(a) apical route	8
(b) femoral route	27
Total patients after BA	9
Total patients with DM	21 (47.7%)
(a) on insulin therapy	9
(b) on peroral antidiabetics	3
(c) DM controlled by diet	9
Total cholesterol (mmol/L)	4.16 ± 1.17
HDL-C (mmol/L)	1.25 ± 0.44
LDL-C (mmol/L)	2.48 ± 0.96
Triglycerides (mmol/L)	1.36 ± 0.66
CRP (mg/L)	20.8 [0.5–75.1]
Lp-PLA_2_ (ng/mL)	1320 ± 342
(a) Lp-PLA_2_ on TAVI group	1296 ± 358
(b) Lp-PLA_2_ on BA group	1413 ± 268

Measurements are expressed as mean ± SD or as total number, with the exception of CRP (median [range]). BP: blood pressure, TAVI: transcatheter aortic valve implantation, DM: diabetes mellitus, HDL-C: high-density lipoprotein concentration, LDL-C: low-density lipoprotein concentration, CRP: C-reactive protein, Lp-PLA_2_: lipoprotein-associated phospholipase A_2_, and BA: balloon angioplasty.

**Table 2:** Pearson correlation between Lp-PLA_2_ and other parameters.

Parameters	Coefficient	*P*
Age	−0.14	NS
Total cholesterol	0.35	0.04
HDL-C	−0.14	0.41
LDL-C	0.40	0.01
Total cholesterol to HDL-C ratio	0.37	0.03
LDL-C to HDL-C ratio	0.36	0.03
Triglycerides	0.43	0.01
BMI	−0.30	0.87
